# β-cell mitochondria in diabetes mellitus: a missing puzzle piece in the generation of hPSC-derived pancreatic β-cells?

**DOI:** 10.1186/s12967-022-03327-5

**Published:** 2022-04-09

**Authors:** Abdoulaye Diane, Noora Ali Al-Shukri, Razik Bin Abdul Mu-u-min, Heba H. Al-Siddiqi

**Affiliations:** Diabetes Research Center, Qatar Biomedical Research Institute (QBRI), Hamad Bin Khalifa University (HBKU), Qatar Foundation (QF), Doha, Qatar

**Keywords:** β-cell, Pancreas, Mitochondria, Diabetes mellitus, hPSC-derived β-cells, Differentiation, Glucose-stimulated insulin secretion (GSIS)

## Abstract

Diabetes mellitus (DM), currently affecting 463 million people worldwide is a chronic disease characterized by impaired glucose metabolism resulting from the loss or dysfunction of pancreatic β-cells with the former preponderating in type 1 diabetes (T1DM) and the latter in type 2 diabetes (T2DM). Because impaired insulin secretion due to dysfunction or loss of pancreatic β-cells underlies different types of diabetes, research has focused its effort towards the generation of pancreatic β-cells from human pluripotent stem cell (hPSC) as a potential source of cells to compensate for insulin deficiency. However, many protocols developed to differentiate hPSCs into insulin-expressing β-cells in vitro have generated hPSC-derived β-cells with either immature phenotype such as impaired glucose-stimulated insulin secretion (GSIS) or a weaker response to GSIS than cadaveric islets. In pancreatic β-cells, mitochondria play a central role in coupling glucose metabolism to insulin exocytosis, thereby ensuring refined control of GSIS. Defects in β-cell mitochondrial metabolism and function impair this metabolic coupling. In the present review, we highlight the role of mitochondria in metabolism secretion coupling in the β-cells and summarize the evidence accumulated for the implication of mitochondria in β-cell dysfunction in DM and consequently, how targeting mitochondria function might be a new and interesting strategy to further perfect the differentiation protocol for generation of mature and functional hPSC-derived β-cells with GSIS profile similar to human cadaveric islets for drug screening or potentially for cell therapy.

## Introduction

Diabetes mellitus (DM) is a chronic disease characterized by impaired glucose metabolism resulting from an absolute or relative insulin deficiency. The incidence and prevalence rates of DM are increasing sharply globally. While there are many different types of diabetes, the two major types of DM are type 1 diabetes (T1DM) and type 2 diabetes (T2DM). T1DM is characterized by selective autoimmune destruction of insulin-producing β-cells within the endocrine pancreas, the islets of Langerhans, whereas the more common type, T2DM results from peripheral tissue insulin resistance and β-cell dysfunction [1–3]. According to the 2019 statistics from the International Diabetes Federation (www.idf.org), the worldwide estimation of adult population with DM was 463 million and this number is projected to increase in the coming 25 years reaching approximatively 700 million people by 2040 (IDF Diabetes Atlas 9th edition www.diabetesatlas.org). About 1 in 11 adults worldwide now have DM, 10% of whom have T1DM [4]. More importantly, the microvascular (retinopathy, nephropathy, neuropathy) and macrovascular (coronary heart disease, myocardial infarction, and stroke) complications associated with DM are extremely costly and difficult to manage, representing a major social, medical and financial challenge for both western and developing countries [5–9]. Current pharmacological treatments for T1DM mostly rely on daily exogenous insulin injections to control glycemia or whole pancreas and islet cell transplantation as an alternative [10]. The islet transplantation approach is circumscribed by a serious scarcity of donor tissues and a potential risk of tissue rejection [11]. Generation of transplantable human β-cells from human induced pluripotent stem cells (hiPSCs) is a future goal of stem cell therapeutics. Moreover, hiPSC-derived β-cells from patients with DM are also critical to gain a better understanding of the disease and its progression [12]. To reach that goal, there is massive effort to efficiently and reproducibly differentiate hPSCs into insulin-expressing β-cells using multi-stages directed differentiation protocols that recapitulate the specific stages of pancreas development. Nevertheless, differentiation attempts do not always result in functional insulin-expressing β-cells instead the differented β-cells show immature phenotype with impaired response to glucose stimulated insulin secretion (GSIS). Both in vivo and in vitro studies highlighted key links between mitochondrial activity and ß-cell functionality [13, 14]. Interestingly, functional and morphological impairments of β-cell mitochondria have been associated with insulin secretory defects in diabetic patients as well as in insulin-resistant iPSC [15, 16]. Therefore, defects in mitochondrial function could contribute to immature phenotypes observed in hPSC-derived pancreatic β-cells.

For T2DM, although the magnitude of the health problems caused by the disorder is well recognized [17], the pathogenesis of the disease remains enigmatic. Pioneering work in the 1960s has demonstrated that T2DM evolves when β-cells fail to release appropriate amounts of insulin in response to glucose [18, 19]. Later, subsequent findings have defined T2DM or non-insulin dependent diabetes as a metabolic syndrome characterized by insulin resistance and progressive loss of pancreatic β-cell function or both [20, 21]. Currently, defective insulin secretion emerges as the main culprit pathogenic factor in T2DM [22], changing, accordingly many research approaches on the treatment and management of T2DM. Therefore, a lot of effort is driven towards finding the optimal differentiation protocol for the generation of hPSC-derived insulin-expressing pancreatic β-cells as a potential source of cells for the future.

There is consensus that mitochondrial metabolism is a major determinant of insulin secretion from pancreatic β-cells. More specifically, mitochondria mediate β-cell responses to extracellular glucose by generating ATP and initiating a cascade of events culminating in the release of insulin, and disruption of mitochondrial oxidative metabolism impairs GSIS [23, 24]. Mitochondria also play a critical role in controlling β-cell mass. Thus, mitochondrial dysfunction causes change in mitochondrial membrane potential, which leads to mitochondria-mediated apoptosis [25]. Available data suggest that increased apoptosis underlies the loss of β-cell mass observed in islets from both T1DM and T2DM, impacting negatively on insulin secretion [26, 27]. Collectively, all these data suggest mitochondria are main players in impaired insulin secretion. Therefore, it is not surprising that β-cell mitochondria have become an important target for DM research. In the present review, we will first briefly describe the pancreatic islets and β-cells, second illustrate the mitochondria and its role in pancreatic β-cell physiology and then provide current insight on the role of mitochondria in β-cell dysfunction in DM and consequently, how targeting mitochondria dynamic and function might be a new and interesting strategy to improve in vitro differentiation into mature and functional hPSC-derived pancreatic β-cells for diabetes therapy.

## What is human pancreatic islet of Langerhans?

In 1869, a medical student Paul Langerhans discovered the existence of clusters of cells in the pancreas, called islets despite their function was unknown [28]. Macroscopically, the pancreas is an unpaired gland of the gastrointestinal tract with an elongated shape, a yellowish-pink aspect and a soft to firm consistency depending on the proportion of fibrosis and fat accumulation in the organ with mixed exocrine–endocrine function. The exocrine cells, representing 98% of the pancreas, release digestive enzymes into the duodenum while the endocrine cells (1–2% of the pancreas) that form clusters of cells called islets of Langerhans, release nutrient-generated hormones into the portal vein. The adult human islets contain four major endocrine cell types: α-cells, β-cells, δ-cells, and γ-cells or pancreatic polypeptide cells and fifth cell type, the Epsilon (ε) or Ghrelin cells that have recently been described [29] each of which releases a different hormone (Table 1). Besides expressed hormones, the different islet cell types can also be distinguished with specific relevant transcription factors [30] (Table 1). The islets of Langerhans play a crucial and vital role in the body because of the scope of this review, we will focus only on β-cells and its produced hormone (i.e. insulin) responsible for maintaining glucose homeostasis. β-cells are the most prominent cell type in the islet. The relative β-cell mass is estimated between 55–75% depending on the morphometric techniques used, the type of samples analyzed [31, 32]. The number of β-cells increases from birth to adulthood [31, 33]. Adult β-cells are heterogenous with mainly two distinct populations identified: the “leader” β-cells representing less than 10% of total β-cells, with pacemaker properties and the ‘follower’ β-cells (> 90%) [34]. β-cells synthesize and secrete insulin, a 51-aminoacid peptide that is essential for cellular nutrient uptake. Insulin has a strong hypoglycemic action and its discovery by Frederick Banting in 1920 has tremendously paved our understanding of diabetes from the ancient Egyptians and its clinical application was one of the major medical breakthroughs of the twentieth century.

**Table 1:** Different cell types in the adult human endocrine pancreatic islet and their characteristics

	Cell types				
	α-cells	β-cells	δ-cells	γ-cells	ε cells
Hormone secreted	Glucagon	Insulin	Somatostatin	Pancreatic polypeptide	Ghrelin
Molecular weight (kDa)	3.5	5.8	1.5	4.2	3.4
Amino acids	29	51	14	36	28
Half-life (min)	8–18	15	1–3	6–7	27–31
Volume % (adult)	30–45	55–75	< 10	< 10	< 1
Specific transcription factors	Nkx2.2, Arx, MafB,	Pdx1, Nkx6.1, MafA, MafB	Pax4, Pdx1,	Nkx2.2, Arx,	?

*Pdx1* pancreatic duodenal homeobox gene 1, *Nkx2.2* NK class of homeodomain-encoding genes 2.2, *Nkx6.1* NK class of homeodomain-encoding genes 6.1, *MafA* V-maf musculoaponeurotic fibrosarcoma oncogene family protein A, *MafB* V-maf musculoaponeurotic fibrosarcoma oncogene family protein B, *Arx* aristaless paired-class homeobox gene, *Pax4* paired homeodomain factor 4, ?: not determined yet

## Structure of mitochondria: a unique and powerhouse of the cell

Mitochondrion is a double-membrane-bound intracellular organelle present in most of the eukaryotic cells. One eukaryotic cell contains hundreds of mitochondria [35]. Mitochondria are termed the powerhouses of the cell as they produce most of the energy or ATP required by the cell. They have their own circular genome (mtDNA) which carries only 37 genes from which 13 genes are coding for proteins of the electron transport chains of the oxidative phosphorylation (OXPHOS) and the rest are coding for the 2 rRNA and the 22 tRNA [36]. Mitochondrial DNA is maternally inherited and replicated independently of the host genome but the great majority of the proteins regulating mitochondrial structure, biogenesis and function are encoded by the nuclear genome and imported into the mitochondria. For example, the transcription and replication of mtDNA is regulated by the mitochondrial transcription factor A (TFAM), which is encoded by the nucleus DNA [37], indicating a clear complex and bidirectional regulation between the nucleus and the mitochondria. Mitochondria has diameter of ~ 0.5 to 1 µm, but their size and structure vary considerably both between different cell types and within the cell [38]. Also, the number of mitochondria in a mammalian cell vary widely by organism, tissue, and cell type. For example, a mature red blood cell has no mitochondria [39], whereas some somatic cells (fibroblast cells), contain > 2000 mitochondria [40]. The mitochondrion is structurally composed of four compartments that carry out specialized functions. These compartments include: (i) the outer membrane, which contains a large number of integral protein structures called porins that allows ions and small molecules to freely diffuse, (ii) the intermembranous space, where protons are accumulated and generate an electrochemical gradient, (iii) the inner membrane, which is freely permeable only for oxygen, CO_2_ and H_2_O. It also allows the transport of adenosine triphosphate (ATP) and contains subunit complexes of the electron transport chains; and (iv) the matrix where oxidation of pyruvate and fatty acids and the tricarboxylic acid (TCA) cycle occur. The presence of cristae formed by infoldings of the inner membrane gives mitochondria its characteristic morphology. Mitochondria are highly dynamic organelles, and their morphology is regulated by cycles of fusion and fission, collectively termed mitochondrial dynamics [41]. Mitochondria serve a number of roles in different cellular processes, with the most significant one of which is that of energetic powerhouses for cellular activities [42].

## Consensus model of mitochondria and glycolysis cooperation in glucose-stimulated insulin secretion

Glucose is an essential metabolic substrate and a major source of energy for almost all mammalian cells including pancreatic β-cells. Pancreatic β-cells sense changes in blood glucose and other secretagogues such as neurotransmitters and circulating hormones and adjust insulin secretion according to the needs of our bodies.

**Fig. 1:**
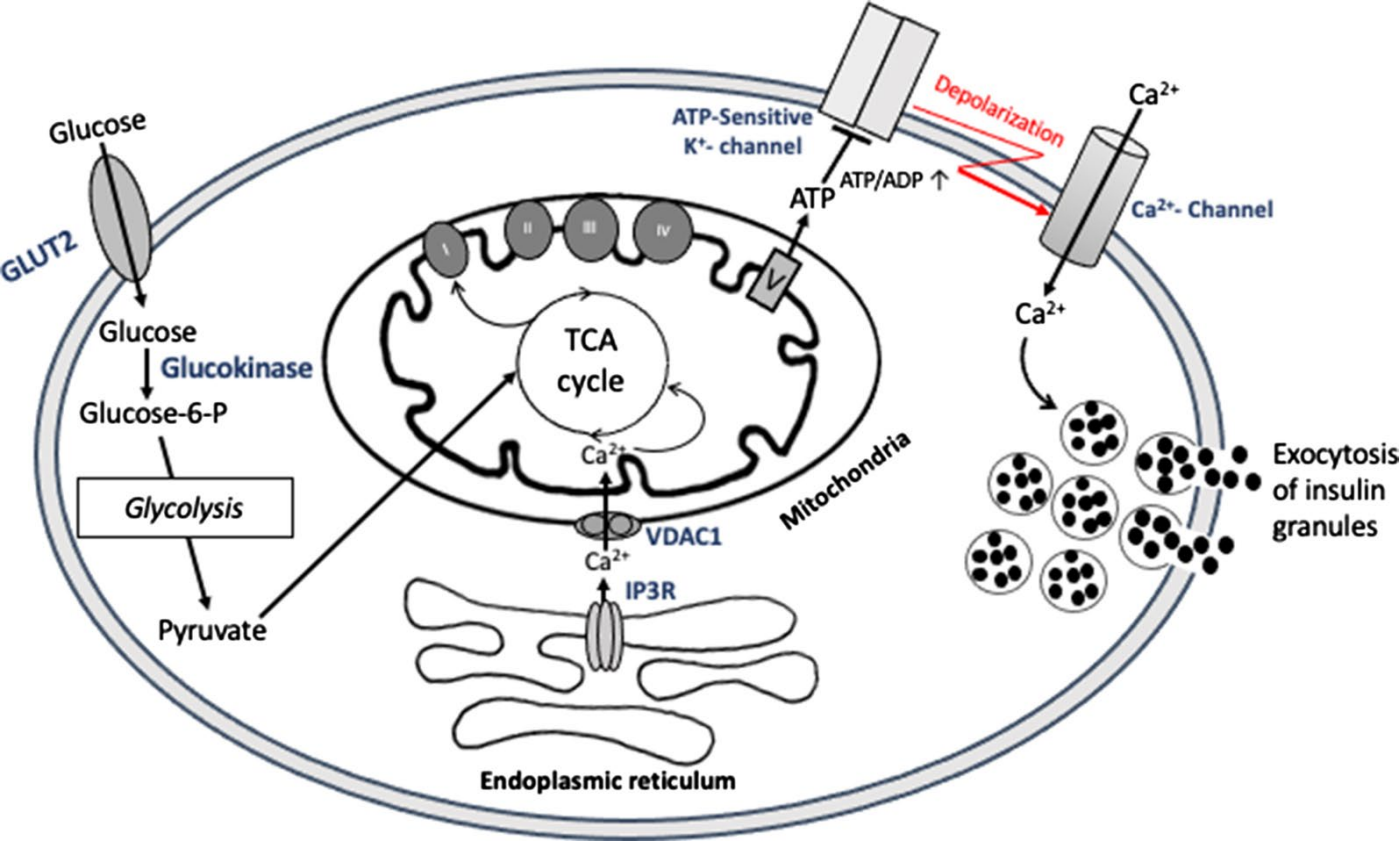
Schematic overview of the consensus model of mitochondria and glycolysis cooperation in glucose-stimulated insulin secretion (GSIS). Glucose is phosphorylated by glucokinase and converted to pyruvate by glycolysis. Pyruvate preferentially enters the mitochondria and fuels the TCA cycle, resulting in the transfer of reducing equivalents to the respiratory chain, leading to hyperpolarization of the mitochondrial membrane (ΔΨm↑) and generation of ATP. Subsequently, closure of KATP- channels depolarizes the cell membrane (ΔΨc↓). This opens voltage-gated Ca^2+^ channels, raising the cytosolic Ca^2+^ concentration, which triggers insulin exocytosis. ER-mitochondria interaction has been proposed to participate in the metabolism–secretion coupling. Ca2^+^ is transferred from ER to mitochondria through inositol 1,4,5-trisphosphate receptor (IP3R) and voltage-dependent anion channel 1 (VDAC1)

**Fig. 2:**
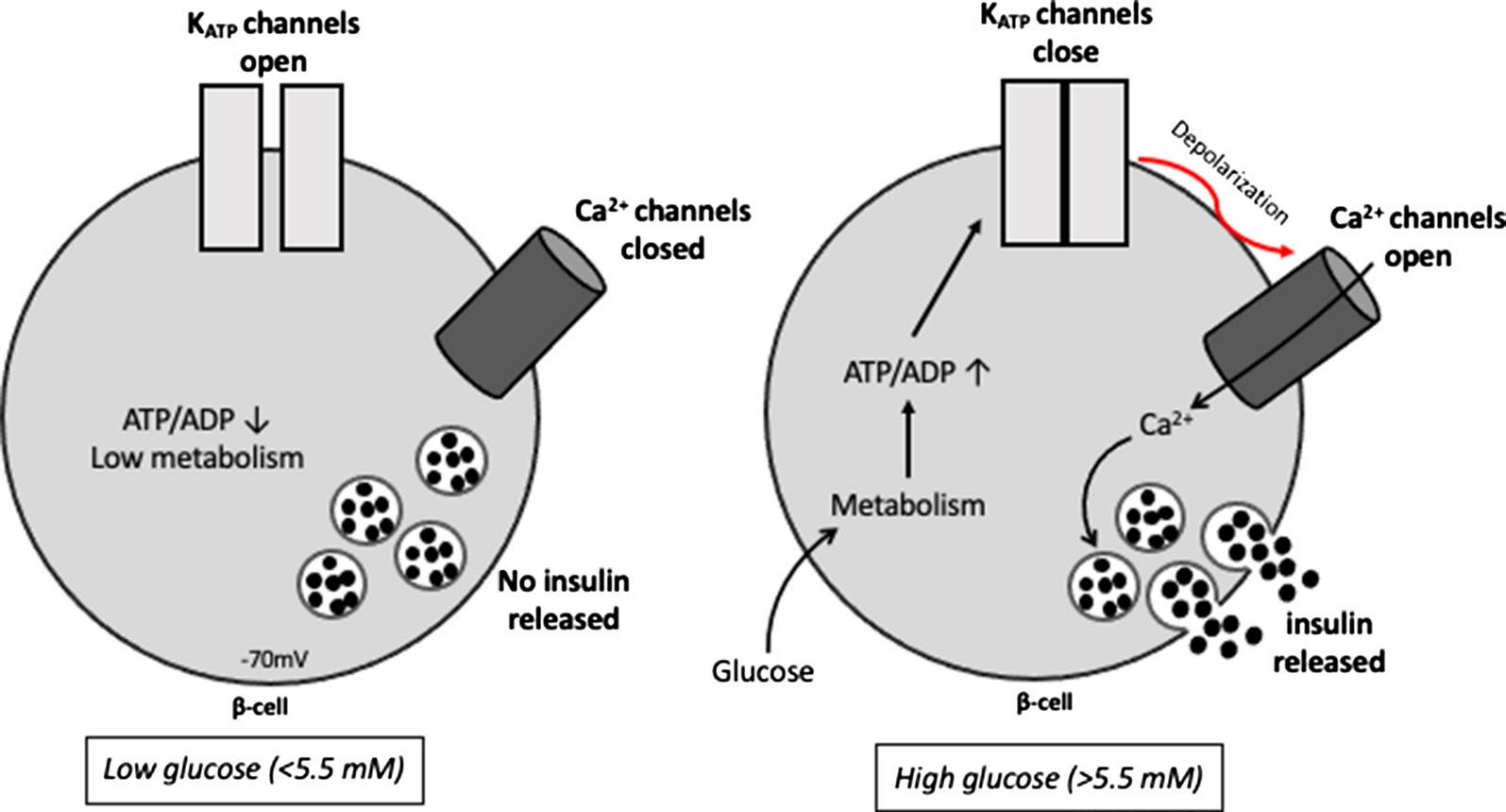
Overview of low glucose and high glucose-stimulated insulin secretion. At basal levels of blood glucose (left panel), the ATP-sensitive K^+^ channels (K_ATP_ channels) in pancreatic β-cells remain open, maintaining membrane hyperpolarization, Ca^2+^ channel closure and inhibiting insulin secretion. A rise in blood glucose (right panel) induces oxidative phosphorylation and ATP production, resulting in the closure of K_ATP_ channels, plasma membrane depolarization, calcium influx leading to increased cytosolic Ca^2+^ that triggers insulin exocytosis: a so-called classical K_ATP_ channel-dependent pathway adapted from Cantley et al. [112]

In healthy β-cells, glucose sensing is largely controlled by the activity of glucokinase [43] and mitochondrial oxidative ATP production [44, 45]. Therefore, a tight coupling between glucose metabolism and insulin exocytosis is required to physiologically modulate the secretory response. Ultrastructural examination of the β-cell has suggested that the mitochondria are often in close proximity to the secretory insulin granules that may facilitate metabolism–secretion coupling [46]. Glucose enters β-cells by facilitated diffusion through the glucose transporter (GLUT2 in rodents; mainly GLUT1 in humans) and is retained inside the cell through its phosphorylation by glucokinase, thereby initiating glycolysis [47] with pyruvate as the main end product due to extremely low lactate dehydrogenase activity in β-cells [48], as opposed to most tissues. Pyruvate is imported into mitochondria, where it feeds the tricarboxylic acid (TCA) cycle. TCA cycle activation induces transfer of electrons from TCA cycle intermediates to the respiratory chain via NADH and FADH_2_ and subsequently ATP production via oxidative phosphorylation (OXPHOS) which leads to increase in cytosolic ATP/ADP ratio. This closes the ATP-sensitive K^+^ channels, producing a membrane depolarization that opens voltage-dependent Ca^2+^ channels: the influx of calcium increases cytosolic Ca^2+^ that triggers insulin exocytosis (Fig. 1). This so-called classical K_ATP_ channel-dependent pathway is the best characterized mechanism underlying the model for coupling of mitochondria and glycolysis cooperation in GSIS in pancreatic β-cells. Furthermore, the paramount role of mitochondria in GSIS is demonstrated by the substantial positive correlation between mitochondrial membrane potential (ΔΨ_M_) and GSIS [49] as well as by complete inhibition of GSIS when OXPHOS is suppressed [50]. Interestingly, evidence has shown that at a subthreshold glucose level, the β-cell K_ATP_ channel is open leading to hyperpolarization of cell membrane and closure of voltage-gated Ca^2+^ channels, preventing thus insulin secretion [51]. In response to a high blood glucose concentration, β-cell K_ATP_ channel-dependent insulin exocytotic machinery is initiated (Fig. 2). Evidence has indicated that glucose can amplify insulin secretion independently of K_ATP_ channels [52]. Molecules involved in the K_ATP_ channel-independent stimulation of insulin secretion are Reactive Oxygen Species (ROS), glutamate, citrate and malate, and cAMP, NADPH, long chain acyl-CoA derivatives [53–56]. These molecules called metabolic coupling factors cannot induce insulin secretion by themselves but are thought to amplify insulin secretion. Their role in the elevation of cytosolic Ca^2+^ goes beyond the generation of ATP necessary for GSIS. Moreover, in the absence of glucose, fatty acids may be metabolized to generate ATP and maintain basal levels of insulin secretion [57, 58]. Fatty acids appear to freely diffuse into pancreatic β-cells through the plasma membrane [59] where they are transformed in long-chain acyl-CoA, by acyl-CoA synthase (ACS), and enter the mitochondria via Carnitine Palmitoyl Transferase 1 (CPT-1) for β-oxidation [59]. The resulting acetyl-CoA is subsequently oxidized in the TCA cycle to generate ATP sufficient for β-cell survival, and basal insulin secretion. When the extracellular glucose concentration is increased, fatty acid oxidation is inhibited, glycolysis takes place [60, 61]. However, the effects of fatty acids on GSIS are directly correlated with chain length and the degree of unsaturation, where long-chain fatty acids (such as palmitate or linoleate) acutely improve, but chronically reduce insulin release in response to glucose stimulation.

## β-cell mitochondria in diabetes mellitus

Mitochondrial dysfunction is an important contributor to human pathology and it is estimated that mutations of mitochondrial DNA (mtDNA) cause approximately up to 1% of all types of diabetes mellitus [62], and often unrecognized by clinicians. In pancreatic β-cells, mitochondria play a central role in coupling glucose metabolism to insulin exocytosis, thereby ensuring strict control of GSIS. Therefore, any defects in mitochondrial function impair this metabolic coupling, and ultimately promote DM. Interestingly, functional and morphological impairments of β-cell mitochondria have been implicated as a key factor in insulin secretory defects associated with DM [15]. In INS-1 cells, exposure to 2 μM of inorganic arsenic, one of the most widespread environmental diabetogens according to epidemiological studies, diminishes GSIS by inhibiting oxygen-dependent mitochondrial metabolism without altering non-mitochondrial respiration [63]. A pivotal role of mitochondria in the pathogenesis of DM is supported by the findings that mtDNA mutations in humans, as well as pancreatic β-cell–specific knockout of TFAM in mouse, cause diabetes [62, 64]. Interestingly, ex-vivo physiological studies of metabolism secretion coupling performed in islets isolated from 7–9-week old TFAM-mutant mice showed reduced hyperpolarization of the mitochondrial membrane potential (ΔΨ_M_), impaired Ca^2+^ signaling and lowered GSIS [62]. Moreover, mouse pancreatic β-cell line, MIN6 with mtDNA depletion are glucose unresponsive, displayed impaired insulin secretion induced by glucose, leucine, and sulfonylureas and are characterized by defective mitochondria [65–67] indicating the involvement of the mitochondrial dysfunction induced by depleted mtDNA in impaired GSIS. Indeed, the impact of mitochondrial DNA mutations is most pronounced in tissues with a low mitotic rate and high ATP production, such as islet cells. Pathophysiologically, there are in total 54 known mtDNA mutations (deletions, substitutions and point mutations) implicated in diabetes development [68]. Among these, the most frequently encountered is the A to G substitution at position bp3243 (A3243G) in a gene encoding for tRNA of leucine [69]. Patients with this mutation have impaired GSIS. The disease is maternally inherited due to the transmission mode of mtDNA affecting approximately 1% of the general population [69] and up to 10% in T1DM patients [70]. In spite the mechanism underlying the A3243G mutation-induced maternal diabetes is not fully understood, most studies pointed out that β-cell dysfunction and impaired insulin secretion become the most prominent characteristics than the insulin resistance in these patients [71]. Moreover, A3243G point mutation results in decreased O_2_ consumption and ATP generation associated with altered mitochondrial morphology [72]. Furthermore, point mutations of the glucokinase (GCK) gene leads to Type 2 Maturity Onset Diabetes of the Young (MODY2), a monogenic diabetes inherited in an autosomal dominant mode [73]. GCK (hexokinase IV), a glucose sensor expressed in pancreatic β-cells, is a key enzyme in glucose metabolism catalyzing the conversion of glucose to glucose-6-phosphate and thus controls GSIS. Studies have reported that diminished β-cell GCK activity is primarily responsible for the hyperglycemia in MODY2 as mice lacking GCK specifically in β-cells are phenotypically similar to animals with a global GCK knock-out [74]. These mice either globally deficient in GCK, or lacking GCK only in β-cells, die within 3 days of birth from severe diabetes. Besides MODY2, MODY3, the most common form of this inherited disease, has been linked to mutations in the transcription factor hepatocyte nuclear factor-1 alpha (HNF1 alpha). HNF1-a controls multiple genes implicated in pancreatic β-cell function and notably in metabolism-secretion coupling. Deletion of the HNF-1a gene in mice results in diabetes [75]. Mechanistically, in β-cells, mutations of HNF-1a gene leads to impaired nutrient-evoked mitochondrial ATP production, mitochondrial membrane potential and reduced intracellular Ca^2+^ and subsequently dysfunctional GSIS [76, 77].

Mitochondria are highly dynamic organelles that function as heterogeneous networks. Their morphology is regulated by coordinated cycles of fusion and fission, collectively referred as “mitochondrial dynamics” [78]. Proteins that regulate mitochondrial fusion and fission have been identified. In mammals, fusion is regulated mainly by mitofusin 1 (Mfn1), mitofusin 2 (Mfn2), and optic atrophy protein 1 (Opa1) while fission is mediated by Fis1, Drp1 and mitochondrial membrane fission factor (MFF) [79–81]. Mitochondrial dynamics have been reported to contribute to mitochondrial quality control and function in a number of systems including pancreatic β-cells [82, 83]. The implication of mitochondrial dynamics in maintaining β-cell mitochondrial homeostasis resulted from study showing impaired mitochondrial morphology in animal models of diabetes [54]. Recent data suggest that β-cells normally contain a filamentous network of mitochondria, but when mitochondria become chronically fused or fragmented, GSIS is impaired [84] [85]. Additionally, abnormal mitochondrial dynamic was observed in pancreatic β-cells postmortem from T2D patients [15]. The role of mitochondrial dynamics proteins in the regulation of β-cell function has been investigated by using genetic tools in β-cell lines [86]. Thus, overexpression of Drp1 or Fis1 in INS-1 cells reduced GSIS [85, 87]. In spite the molecular mechanisms remain not fully understood yet, these studies highlighted the pivotal role on how changes in mitochondrial dynamics and morphology affect pathways that influence pancreatic β-cells insulin secretory function. Understanding the molecular mechanisms controlling mitochondrial dynamics is crucial to decipher how mitochondrial shape meets or correlates with β-cell function.

## ER-mitochondria communication in the control of GSIS

Inter-organelle communication is an emerging aspect of cell biology, and the nature of this network has been reported to allow the adaptations of metabolism according to cellular needs. Importantly, ER and mitochondria are no longer considered as individual organelles in the cell as they physically interact in a highly dynamic and regulated manner, forming specific microdomains, termed mitochondria-associated membranes (MAM) [88]. Organelle contact site does not involve membrane fusion but is mediated through “protein bridges” [89]. It is well established that MAM play a central role in cellular Ca^2+^ homeostasis [90, 91] and more recently, ER-mitochondria interactions have been shown to potentially regulate several aspects of mitochondria functions, including mitochondria dynamics, oxidative metabolism [91, 92] and apoptosis [93]. Most of these functions are intimately connected to the Ca^2+^ status of mitochondria, since Ca^2+^ transferred from ER to mitochondria is pivotal for the regulation of mitochondrial energy metabolism (as three enzymes of TCA cycle are Ca^2+^ dependent), thus influencing ATP synthesis: the inhibition, or the absence of the transfer causes a decrease in ATP levels [94]; therefore, MAM could be viewed as an important hub for hormonal and nutrient signalling in many organs. Until the last decades, the most described functions of ER-mitochondria interactions are lipid biosynthesis, pointing at a role of MAM in lipid metabolism [95–97]. Importantly, alterations of the ER-mitochondria coupling leading to disruption of lipid homeostasis have been commonly associated with the pathogenesis of metabolism-related diseases such as obesity, T2DM, non-alcoholic fatty liver disease (NAFLD) [98–100]. This subcellular communication has now been integrated to the involvement of ER and mitochondria in DM. In this regard, recent evidence reports an association between ER-mitochondria miscommunication and β-cell dysfunction in diabetic patients [101]. Mechanistically, ER-mitochondria miscommunication promotes mitochondrial dysfunction, ER stress, altered Ca^2+^ homeostasis and subsequently leads to alterations of both insulin action and secretion in DM [102]. Furthermore, induction of ER stress by treatment with palmitate significantly reduced ER-mitochondria crosstalk and altered GSIS in the murine Min6-B1 β-cell line [101]. Collectively, this evidence highlights the importance of MAM in nutrient-regulated signaling pathways in the control of glucose and insulin homeostasis and suggests that targeting MAM structure and function could be a novel strategy for the management of DM and generation of fully functional hPSC-derived β-cells.

## Role of mitochondria in the functionality of hiPSC-derived pancreatic cells

β-cells are known to facilitate glucose-stimulated insulin secretion (GSIS) through increased mitochondrial oxidative ATP production [54, 103], indicating that increased mitochondrial activity is a cellular component required for GSIS. Whereas the role of pancreatic β-cell mitochondria in impaired GSIS and DM is well established, its role in the maturation and functionality of hPSC-derived pancreatic β-cells remains poorly understood. Current pharmacological treatments for T1DM mostly rely on daily injections of exogenous insulin to control glycemia or whole pancreas and islet cell transplantation as an alternative [10]. The islet transplantation approach is circumscribed by a serious scarcity of donor tissues and a potential risk of tissue rejection [11]. Generation of transplantable human β-cells from human pluripotent stem cells, such as embryonic stem cells (hESC) and induced pluripotent stem cells (hiPSC), is an ultimate goal of stem cell therapeutics. However, most of hPSC-derived β-cells (insulin-expressing β-cells) generated from in vitro differentiation protocols express known adult β-cell markers but are not fully functionally mature [34]. A large number of studies have found that many transcription factors such as PDX1, NKX6.1, MAFA play important roles in the process of functional maturation of immature β-cells [104]. The absence or abnormal expression of any of these key factors leads to generation of hPSC-derived β-cells that resemble fetal cells with immature phenotype [104]. Functionally, the immaturity of mitochondrial NADH shuttles contributes to the inability of fetal, newborn, and newly regenerated β-cells to secrete insulin in response to glucose [105]. NADH shuttle system is essential for coupling glycolysis with the activation of mitochondrial energy metabolism to trigger GSIS. As mitochondria plays an important and crucial role in β-cells function, it is possible that functionally defective mitochondria NADH shuttle system underlies the generation of immature and non-functional differentiated hPSC-derived β-cells. Also, all insulin-expressing hPSC-derived β-cells obtained in vitro do not fully recapitulate the biphasic insulin secretion in vitro observed with human cadaveric islets. They do not achieve an in vitro GSIS response equivalent to that of cadaveric islets in terms of the magnitude of insulin secretion [106]. In cadaveric islet, the response to glucose challenge was approximately tenfold higher than basal secretion, whereas only 2.2-fold increased was observed in hPSC-derived β-clusters in spite of equal amount of mitochondrial mass per cell [106], suggesting that differentiated hPSC-derived β-cells might have metabolically dysfuntional mitochondria. Interestingly, reduced anaplerotic cycling in the mitochondria has been identified as an underlying mechanism associated with reduced GSIS in hPSC-derived β-cells [106]. Therefore, proper mitochondrial function is a cornerstone of β-cell stimulus secretion coupling. β-cells are known to functionally mature postnatally, including acquiring the ability to properly secrete insulin in response to glucose [107, 108]. Consistently, islets isolated from neonatal mice showed impaired GSIS [109] likely attributed to a generalised low activity and expression of the key metabolic genes including mitochondrial membrane shuttles (glycerol phosphate, malate–aspartate, pyruvate–citrate and pyruvate–malate shuttles), through which glycolysis-derived NADH is reoxidised. Additionally, increasing NADH shuttle activity in fetal rat islets using mitochondrial glyceraldehyde 3-phosphate dehydrogenase (GPDH) cDNA significantly improved GSIS [105]. This indicates that the immaturity of the NADH shuttles contributes to impaired GSIS observed in fetal β-cells. Moreover, induction of ERRγ, one of the three paralogs of the estrogen-related receptors (ERRs), known to drive a transcriptional network activating mitochondrial oxidative phosphorylation, the electron transport chain, and ATP production required for GSIS improved insulin secretion in neonatal islets as well as in iPSC-derived β-like cells [109]. In addition, exenatide, a GLP-1 analog, has been recently reported to enhance GSIS in Friedreich ataxia patient-specific iPSC through its mitochondrial function promoting effect [110]. These results strongly suggest a key role for metabolically active mitochondria in the induction of functional β-cells. Most differentiation protocols [111] that attempt to mimic developmental events used small molecules to either activate or repress key transcriptional factors for lineage specification signals may be insufficient to fully promote differentiation and maturity. Indeed, other factors required for GSIS such as induction of metabolically active mitochondria may also be needed for maturation to achieve functionality of hPSC-derived β-cells in vitro. Collectively, all these findings suggest that targeting mitochondrial function may be the missing puzzle piece to further perfect the differentiation protocol for generation of mature and functional hPSC-derived β-cells with GSIS profile similar to human cadaveric islets for drug screening or potentially for cell therapy in the future.

## Conclusion

Multiple mechanisms are involved in impaired insulin secretion associated with diabetes mellitus due to either dysfunction or loss of pancreatic β-cells. However, evidence suggests that dysfunction in ß-cell mitochondrial metabolism and function contributes to insulin secretory defects in diabetic patients. In this review we highlight the role of mitochondrial ß-cell in coupling glucose metabolism to insulin exocytosis and summarize recent progress for the implication of mitochondria in β-cell dysfunction in defective insulin secretion in DM as well as in generated in vitro hPSC-derived ß-cells with immature phenotype. Therefore, for research focused on developing β-cell replacement strategies for potential diabetes therapy or drug screening, targeting mitochondria function might be a new and interesting approach to further perfect the differentiation protocol for generation of mature and functional hPSC-derived β-cells with GSIS profile similar to human cadaveric islets.

## Data Availability

Not applicable.
